# Risk factors for mortality in prostatic abscess: Insights into patient characteristics and drainage practices

**DOI:** 10.1371/journal.pone.0349673

**Published:** 2026-06-01

**Authors:** Yun-Chen Tsai, Jian-Ri Li, Chiann Yi Hsu, Che-An Tsai

**Affiliations:** 1 Department of Medical Education, Taichung Veterans General Hospital, Taichung, Taiwan; 2 Department of Radiation Oncology, Taipei Medical University-Shuang Ho Hospital, New Taipei City, Taiwan; 3 Department of Urology, Taichung Veterans General Hospital, Taichung, Taiwan; 4 Department of Medical Research, Taichung Veterans General Hospital, Taichung, Taiwan; 5 Division of Infectious Disease, Department of Internal Medicine, Taichung Veterans General Hospital, Taichung, Taiwan; Children’s National Hospital, George Washington University, UNITED STATES OF AMERICA

## Abstract

Prostatic abscess, which is an uncommon urinary tract disease, can lead to mortality if not properly treated. This retrospective study aimed to identify risk factors associated with mortality in patients with prostatic abscess, with the goal of providing insights to improve clinical outcomes. The study was conducted on patients diagnosed with prostatic abscess and hospitalized between January 2007 and December 2021. The diagnosis was confirmed through imaging. Out of 822 hospitalized patients with acute prostatitis or prostatic abscess, 102 had prostatic abscess (12.4%). The mean age was 68.8 years old, and overall mortality was 8.8%. Drainage was performed in 15.7% cases. There was no significant difference in abscess size, prostate volume, or percentage of abscess formation at other sites between survivors and non-survivors. Age and use of alpha blockers were identified factors related to death, and older age remained significantly related to mortality (OR=1.07) in multivariate analysis. Factors associated with drainage or transurethral resection of the prostate were analyzed. Abscess size (OR=1.70), cystostomy (OR=4.56), prostate volume (OR=1.01) and the presence of abscess in other organs (OR=3.89) were associated with drainage, whereas abscess size (OR=1.58), cystostomy (OR=5.13), and liver cirrhosis (OR=5.67) were associated with transurethral resection of prostate. There were no differences between the drainage and non-drainage groups after propensity score matching for age, BMI, and abscess size. In our study, advanced age was the primary factor associated with mortality in patients with prostate abscess. Other factors, including drainage and abscess size, were not significantly associated with mortality; however, these findings may be influenced by confounding factors and the limited number of mortality events, warranting further investigation.

## Introduction

Prostatic abscess is a lower urinary tract infection mostly as one complication of acute prostatitis [1]. In a retrospective review of 142 patients with acute prostatitis having received an image survey for prostate, 31 cases of prostatic abscess were found [2]. Risk factors such as immunodeficiency and urological manipulations were identified in the post-antibiotic era [3,4]. The clinical diagnosis of prostatic abscess can be made upon presence of positive fluctuation with tenderness during digital rectal examination, with image confirmation by transrectal ultrasound (TRUS), computed tomography (CT), or magnetic resonance imaging (MRI) [4–7]. It is suggested that imaging be performed in patients who do not respond to antibiotics within 48 hours to distinguish between acute prostatitis and prostatic abscess [8]. Treatments of prostatic abscess typically involve conservative antibiotics treatment, transperineal- or TRUS-guided aspiration and transurethral resection of prostate abscess (TURP). While aspiration offers a less invasive approach, transurethral drainage is associated with a lower recurrence rate and should therefore be considered for more complex abscesses [9]. A recent retrospective study observed that a single abscess of smaller size (<2.2 cm) and a smaller prostate volume are factors determining candidacy for the conservative treatment [10].

The mortality rate of prostatic abscess varies in current studies, ranging from 0% to 25%, with sepsis being the most commonly identified cause of death [7,9–14]. Despite the declining incidence and mortality rate after the extensive use of antibiotics, management of prostatic abscess still presents a challenging situation due to lack of evidence-based guidelines [15]. Currently, most published studies on prostatic abscess mainly reflect on outcomes of different interventions [14]. Insufficient data on patients receiving only medical treatment may lead to incomplete evaluation of prostatic abscess and its outcomes. This retrospective study thus aimed to investigate patients with prostatic abscess and to identify risk factors that affect mortality in a single medical center. Hopefully to provide more information on optimizing the outcome of prostatic abscess.

## Materials and methods

### Database

The database was constructed in a web-based format including all clinical information of patients who received medical care in a single medical institute since January 2000. It contained de-identified data on diagnosis, English-based charts, and lab examinations from outpatient visits, hospitalizations, and emergency department admissions.

### Patient selection

Between January 1, 2007 and December 31, 2021, patients 20 years of age or older who were diagnosed with prostatic abscess or acute prostatitis and hospitalized were included. Data was collected without information that could identify individual participants. In the ICD-9 system, prostatic abscess and acute prostatitis were both assigned to code 601. The medical chart and image (including transabdominal ultrasound, TRUS and CT) were further reviewed to confirm the diagnosis of prostate abscess. Image diagnosis of prostatic abscess was based on presence of non-enhancing irregular fluid collection with thick wall or septation in prostate [16,17]. The exclusion criteria included previously diagnosed prostate cancer or cancer metastasis to the prostate.

### Study assessment

This study was conducted through retrospective clinical data claiming, and was approved by the institute review board of Taichung Veterans General Hospital (CE22411B) with patient consents waived. The data was accessed for research purposes on February 14, 2023.

**Fig 1:**
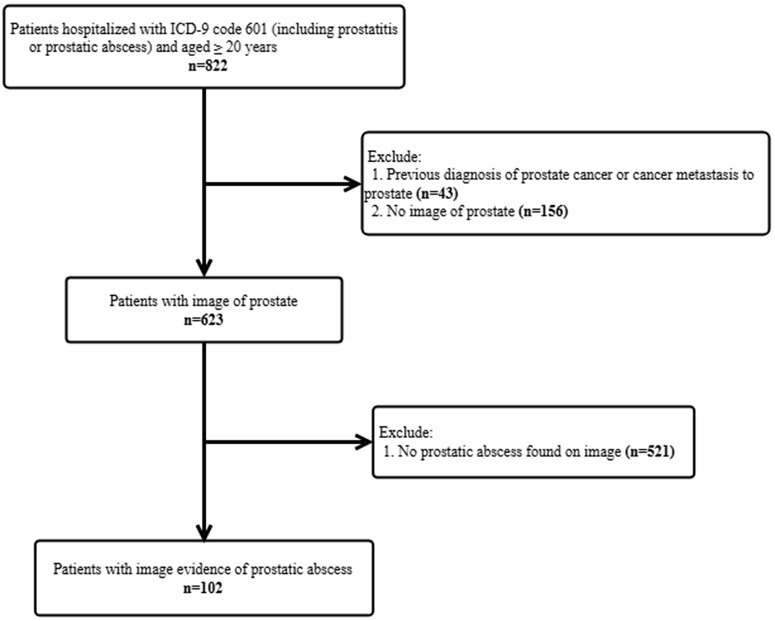
Prostatic abscess patient selection. The figure showed the selection process from 822 patients to 102 cases of prostatic abscess.

Data tabulated from medical charts included age, body mass index, initial vital signs at admission, admission source (emergency room or not), admitting department (urology or non-urology), and initial laboratory results following admission, including white blood cell count, CRP, blood culture and urine culture, urinary tract lithotripsy or TURP three months before admission, comorbidities (hypertension, ICD9 401–405; diabetes mellitus, ICD9 250; ischemic heart disease ICD9 410–414; cerebrovascular disease,ICD9 430–438; hyperlipidemia ICD9 272; chronic obstructive pulmonary disease ICD9 490–496; chronic kidney disease (COPD) ICD9 585), regular (>14 days) benign prostatic hyperplasia medication use (alpha blocker and 5-alpha reductase) before admission, prostate volume, foley insertion or cystostomy during admission, prostate abscess drainage (TRUS-guided aspiration, transperineal ultrasound-guided aspiration, and transurethral resection of prostatic abscess) during admission, death date, admission and discharge date. TURP was done under general or regional anesthesia and CT guided drainage was done with local anesthesia. Prostatic abscess size (cm), presence of abscess at other sites were reviewed and measured based on CT images or ultrasonography. Patients were classified into the drainage group if they had received drainage during this admission. All the continuous variables are displayed as mean and categorical variables as percentages. The study flow chart is listed in Fig 1.

### Statistical analysis

The Mann-Whitney U-test was applied for the continuous variables, such as age, lab examinations, prostate volume, abscess size, and hospital stay length. The Chi-square test and Fisher’s t-test were used for categorical variables, such as comorbidities, urinary tract intervention before admission, abscess drainage during admission and mortality. Univariate logistic regression analyses were performed to evaluate the association between each variable and the outcome. At most two variables were selected for multivariate analysis based on clinical relevance and potential confounding effects due to the limited number of outcome events. Propensity score matching analysis was performed to minimize selection bias between patients who underwent drainage and those who did not. Patients in the drainage and non-drainage groups were matched in a 1:1 nearest-neighbor fashion without replacement. Covariate balance after matching was assessed using standardized mean differences (SMD), with an SMD < 0.1 indicating acceptable balance. A two-sided p value < 0.05 was considered statistically significant. Missing data was managed using the available case analysis approach. All statistical analyses were performed using SAS software version 9.2 (SAS Institute, Inc., Cary, NC, USA).

## Results

There were 822 patients diagnosed with acute prostatitis or prostatic abscess and received in-hospital treatment in our database. Forty-three patients were excluded due to a prior diagnosis of prostate cancer or metastatic disease. An additional 156 patients were excluded because of unavailable imaging data, and 521 patients were excluded because no radiologic evidence of prostatic abscess was identified (including 3 patients with identified pus during TURP procedure). After exclusion, a total of 102 cases had image-proved abscess. Of the 102 patients studied, 9 died and 93 survived. The overall mortality rate was 8.8%. The mean age of the patients diagnosed with prostate abscess was 68.8 ± 14.1 years. Older age was observed in the mortality group (mean age 85.1 ± 11.2 years) compared with the survival group (mean age 67.2 ± 13.4 years) (p < 0.001). Hypertension (55.9%) and diabetes mellitus (48.0%) were two of the most common comorbidities identified at admission. There was no significant difference in body mass index, comorbidities such as hypertension, diabetes mellitus, hyperlipidemia, chronic kidney disease, COPD, liver cirrhosis, ischemic heart disease, and cerebrovascular disease. Mean prostate volume was 54.7 ± 35.1 cm^3^, and the volume didn’t vary between 2 groups (p = 0.989). Twenty-six patients (25.5%) regularly took alpha blockers before admission, and only 1 person (1.0%) took 5 alpha reductase inhibitors. Additionally, prior use of alpha blockers was markedly more common among patients who died, with 66.7% reporting alpha blocker use before admission compared with 21.5% among survivors (p = 0.008). One patient received lithotripsy due to ureter-pelvic junction stone, and 2 people underwent transurethral resection of prostate within 3 months of admission. (Table 1). None of them expired during this admission.

**Table 1:** Patient characteristics.

	Total (n = 102)	Survivors (n = 93)	Non-Survivors (n = 9)	p value
Age, years Mean (SD)	68.8 (14.1)	67.2 (13.4)	85.1 (11.2)	<0.001**
BMI, kg/m^2^ Mean (SD)	24.2 (4.0)	24.3 (4.0)	21.7 (2.8)	0.266
Comorbidities				
CKD (%)	30 (29.4%)	25 (26.9%)	5 (55.6%)	0.119
COPD (%)	23 (22.5%)	19 (20.4%)	4 (44.4%)	0.113
Cerebrovascular disease (%)	30 (29.4%)	28 (30.1%)	2 (22.2%)	1.000
Diabetes mellitus (%)	49 (48.0%)	45 (48.4%)	4 (44.4%)	1.000
Hyperlipidemia (%)	25 (24.5%)	21 (22.6%)	4 (44.4%)	0.217
Hypertension (%)	57 (55.9%)	50 (53.8%)	7 (77.8%)	0.292
Ischemic heart disease (%)	24 (23.5%)	21 (22.6%)	3 (33.3%)	0.436
Liver cirrhosis (%)	8 (7.8%)	8 (8.6%)	0 (0%)	1.000
Prostate volume, cm^3^ Mean (SD)	54.7 (35.1)	54.3 (34.7)	60.0 (42.4)	0.989
Alpha blocker (%)	26 (25.5%)	20 (21.5%)	6 (66.7%)	0.008**
5-alpha reductase inhibitor (%)	1 (1.0%)	1 (1.1%)	0 (0%)	1.000
Procedure within 3 months before admission				
Lithotripsy (%)	1 (1.0%)	1 (1.1%)	0 (0%)	1.000
TURP (%)	2 (2.0%)	2 (2.2%)	0 (0%)	1.000

The table presents the characteristics of patients diagnosed with prostatic abscess, divided into survival and non-survival groups.

Mann-Whitney U-test. Chi-Square test. Fisher’s exact test. *p < 0.05, **p < 0.01. Continuous data are expressed as mean (standard deviation). Categorical data are expressed as number and percentage. BMI, body mass index; COPD, chronic obstructive pulmonary disease; TURP, transurethral resection of prostate.

Regarding the first body temperature recorded during admission, half patients (55.9%) had fever or hypothermia, as defined as body temperature < 36.0°C or > 37.9°C. More percentage (88.9%) of patients who died eventually had fever at first measurement. As to first examined lab data, there was no statistical difference of white blood cell count or CRP between two groups. Two-thirds patients (68.6%) had positive urine cultures, and 39.2% cases were with positive blood cultures.

Most patients (96.1%) had received CT image, and were diagnosed prostate abscess accordingly. Other diagnostic methods included transabdominal echo (2.0%) and transrectal echo (2.0%). There was no difference among diagnostic methods between survivors and non-survivors. Eighty-nine out of 93 cases (95.7%) in the survival group and all patients in the non-survival group had CT image. There were no statistically significant differences between survivors and non-survivors in mean abscess size (3.1 ± 1.4 cm vs. 2.6 ± 1.3 cm, p = 0.275) or abscess-to-prostate ratio (0.07 ± 0.04 vs. 0.05 ± 0.02, p = 0.164). Abscess involving other regions were identified in 14 patients (13.7%), with 12 (12.9%) in the survival group and 2 (22.2%) in the non-survival group (p = 0.607). The most frequent site of abscess was liver (50%) and the second was kidney (28.5%). Other sites included para-spinal region, seminal vesicle, gluteal muscle, pelvic wall and Fournier’s gangrene. Of the two patients who died, one was with ruptured liver abscess, and another with liver and para-spinal abscess. (Table 2).

**Table 2:** Examination and management during admission.

	Total N = 102	Survivors N = 93	Non-Survivors N = 9	P value
Fever or hypothermia (%)	57 (55.9%)	49 (52.7%)	8 (88.9%)	0.074
WBC,/µL Mean (SD)	15778.7 (8066.5)	15762.2 (7991.6)	15950.0 (9328.0)	0.883
CRP, mg/dL Mean (SD)	15.4 (10.1)	15.1 (10.0)	17.8 (11.7)	0.483
Urine culture positive (%)	70 (68.6%)	65 (69.9%)	5 (55.6%)	0.456
Blood culture positive (%)	40 (39.2%)	35 (37.6%)	5 (55.6%)	0.309
Diagnostic method				
CT (%)	98 (96.1%)	89 (95.7%)	9 (100%)	1.000
Transabdominal ultrasound (%)	2 (2.0%)	2 (2.2%)	0 (0%)	
Transrectal ultrasound (%)	2 (2.0%)	2 (2.2%)	0 (0%)	
Abscess size, cm Mean (SD)	3.1 (1.4)	3.1 (1.4)	2.6 (1.3)	0.275
Abscess-prostate ratio,/cm^2^ Mean (SD)	0.07 (0.04)	0.07 (0.04)	0.05 (0.02)	0.164
Abscess at other organs (%)	14 (13.7%)	12 (12.9%)	2 (22.2%)	0.607
Foley insertion (%)	55 (53.9%)	50 (53.8%)	5 (55.6%)	1.000
Cystostomy (%)	16 (15.7%)	16 (17.2%)	0 (0%)	0.348
Drainage (%)	16 (15.7%)	16 (17.2%)	0 (0%)	0.348
Length of stay, days Mean (SD)	22.1 (18.4)	22.5 (17.9)	18.0 (23.9)	0.078

This table presents the differences in lab examinations, abscess size, interventions and hospital stay between survivors and non-survivors.

Mann-Whitney U-test. Chi-Square test. Fisher’s exact test. *p < 0.05, **p < 0.01. Continuous data are expressed as mean (standard deviation). Categorical data are expressed as number and percentage. WBC, white blood cell count; CRP, c-reactive protein; CT, computed tomography.

Foley catheter insertion was done in 55 cases (53.9%). There were 16 patients who received cystostomy, all of them being in the survival group. Drainage was performed in 16 cases (15.7%) of prostatic abscess, all in the survival group. Average hospital stay length was 22.1 ± 18.4 days. There was no statistically significant difference in hospital stay between patients who died and survivors (18.0 ± 23.9 vs. 22.5 ± 17.9 days, p = 0.078). (Table 2).

Univariate analysis of risk factors for prostate abscess-related death showed significant correlation with age (OR 1.18, p = 0.002) and alpha blockers use (OR 7.30, p = 0.008). After adjustment for age and alpha blockers use in the multivariate analysis, only age remained significantly associated with prostate abscess related mortality (OR 1.15, p = 0.008). No other significant factors were identified in risk factor regression for prostatic abscess related-death. (Table 3).

**Table 3:** Risk factor regression for prostate abscess related-death.

	Univariate model			Multivariate model		
	OR	95% CI	*p* value	OR	95% CI	*p* value
Age	1.18	1.06–1.30	0.002**	1.15	1.04–1.28	0.008**
BMI	0.82	0.61–1.11	0.207			
Fever or hypothermia	7.18	0.86–59.75	0.068			
WBC	1.00	1.00–1.00	0.947			
CRP	1.03	0.96–1.10	0.450			
Abscess size	0.75	0.42–1.35	0.340			
Prostate volume	1.00	0.99–1.02	0.659			
Alpha blocker	7.30	1.68–31.80	0.008**	3.37	0.64–17.65	0.151
CKD	3.4	0.84–13.68	0.085			
COPD	3.12	0.76–12.74	0.114			
Cerebrovascular disease	0.66	0.13–3.39	0.622			
Diabetes mellitus	0.85	0.22–3.38	0.821			
Hyperlipidemia	2.74	0.68–11.14	0.158			
Hypertension	3.01	0.59–15.26	0.183			
Ischemic heart disease	1.71	0.39–7.45	0.472			

This table shows risk factors associated with death of patients with prostatic abscess in uni- and multivariate regression.

Logistic regression. *p < 0.05, **p < 0.01. BMI, body mass index; WBC, white blood cell count; CRP, c-reactive protein; CKD, chronic kidney disease; COPD, chronic obstructive pulmonary disease.

Patient characteristics between those who received drainage and those who underwent conservative treatment alone were compared. A univariate analysis was conducted for patients who underwent abscess drainage. Three factors were found to be associated with abscess drainage, including prostate volume (OR 1.01, p = 0.045), size of the prostatic abscess (OR 1.70, p = 0.003), and receiving cystostomy (OR 4.56, p = 0.014). Two variables were selected based on clinical relevance and potential confounding effects for multivariate analysis. Both abscess size (OR 1.65, p = 0.008) and cystostomy (OR 3.64, p = 0.046) remained statistically significant in the multivariate analysis. Of the 16 patients receiving drainage of abscess, 12 had TURP, 3 had CT-guided transperineal aspiration and 1 had suprapubic prostatectomy. Comparing between TURP and non-TURP group, association was found in cystostomy (OR=5.13, p = 0.014), liver cirrhosis (OR=5.67, p = 0.032), and abscess size (OR=1.58, p = 0.019). In multivariate analysis, both abscess size (OR=1.52, p = 0.041) and cystostomy (OR=4.16, p = 0.039) remained statistically significant. (Table 4). Categorical comparisons of variables between TURP and non-TURP, as well as drainage and non-drainage were provided in the supplementary tables (S1 and S2 Tables).

**Table 4:** Regression analysis of factors associated with TURP (n = 12) and drainage (n = 16).

	Univariate model			Multivariate model		
	OR	95% CI	*p* value	OR	95% CI	*p* value
TURP (n = 12)						
Age	0.98	0.94–1.02	0.332			
BMI	1.07	0.92–1.25	0.383			
Fever or hypothermia	1.12	0.33–3.80	0.856			
CRP	1.00	0.94–1.07	0.929			
Abscess size	1.58	1.08–2.31	0.019*	1.52	1.02-2.27	0.041*
Prostate volume	1.01	1.00–1.03	0.085			
Cystostomy	5.13	1.38–19.00	0.014*	4.16	1.08-16.09	0.039*
Diabetes mellitus	2.39	0.67–8.51	0.179			
Hypertension	0.76	0.23–2.55	0.663			
Liver cirrhosis	5.67	1.16–27.72	0.032*			
Drainage (n = 16)						
Age	0.97	0.94–1.01	0.158			
BMI	1.10	0.95–1.26	0.192			
Fever or hypothermia	0.76	0.26–2.20	0.607			
CRP	1.00	0.94–1.06	0.993			
Abscess size	1.70	1.19–2.43	0.003**	1.65	1.14-2.38	0.008**
Abscess at other organs	3.89	1.10–13.75	0.035*			
Prostate volume	1.01	1.00–1.03	0.045*			
Cystostomy	4.56	1.36–15.26	0.014*	3.64	1.02-12.97	0.046*
Diabetes mellitus	2.01	0.67–6.02	0.213			
Hypertension	0.56	0.19–1.64	0.291			
Liver cirrhosis	3.74	0.80–17.55	0.095			

This table shows factors associated with drainage or TURP in patients with prostatic abscess in uni- and multivariate regression.

Logistic regression. *p < 0.05, **p < 0.01. BMI, body mass index. CRP, c-reactive protein.

After 1:1 propensity score matching on age, BMI and size of prostatic abscess, 16 patients in the drainage group were matched with 16 patients in the non-drainage group. Age and abscess size were well balanced between groups (SMD < 0.1), whereas BMI remained imbalanced (SMD = 0.16). One mortality event occurred in the non-drainage group, and none was observed in the drainage group. Variables such as fever or hypothermia, WBC, CRP and length of hospital stay didn’t differ between drainage and non-drainage groups. (Table 5).

**Table 5:** Comparison of drainage and non-drainage groups after 1:1 propensity score matching on age, BMI and abscess size.

	Drainage (n = 16)	Non-Drainage (n = 16)	*p* value	SMD
Age, years Mean (SD)	64.2 (8.6)	65.2 (14.5)	0.395	0.08
BMI, kg/m^2^ Mean (SD)	25.4 (3.7)	24.8 (3.7)	0.330	0.16
Abscess size, cm Mean (SD)	4.1 (1.9)	4 (1.6)	0.874	0.08
Fever or hypothermia (%)	8 (50.0)	8 (50.0)	1.000	<0.001
WBC,/µL Mean (SD)	15041.9 (8937.4)	15696.9 (8584.3)	0.977	0.08
CRP, mg/dL Mean (SD)	15.4 (9.9)	14.2 (10.2)	0.812	0.12
Prostate volume, cm^3^ Mean (SD)	72.5 (49.7)	64.1 (42.5)	0.270	0.18
Alpha blocker (%)	4 (25.0)	4 (25.0)	1.000	<0.001
Cystostomy (%)	6 (37.5)	3 (18.8)	0.433	0.43
Length of stay, days Mean (SD)	20.4 (9.8)	25.9 (24.1)	1.000	0.30
Death (%)	0 (0)	1 (6.3)	1.000	0.37
Comorbidities				
CKD (%)	5 (31.3)	5 (31.3)	1.000	<0.001
COPD (%)	1 (6.3)	4 (25.0)	0.333	0.54
Cerebrovascular disease (%)	5 (31.3)	6 (37.5)	0.710	0.13
Diabetes mellitus (%)	10 (62.5)	8 (50.0)	0.476	0.25
Hyperlipidemia (%)	5 (31.3)	4 (25.0)	1.000	0.14
Hypertension (%)	7 (43.8)	9 (56.3)	0.480	0.25
Ischemic heart disease (%)	4 (25.0)	3 (18.8)	1.000	<0.001
Liver cirrhosis (%)	3 (18.8)	1 (6.3)	0.600	0.39

The table presents the characteristics of patients matched on age, BMI, and abscess size.

Mann-Whitney U-test. Chi-Square test. Fisher’s exact test. Standardized mean differences (SMDs) are shown to assess covariate balance. An SMD < 0.1 is considered indicative of adequate balance. *p < 0.05, **p < 0.01. Continuous data are expressed as mean (standard deviation). Categorical data are expressed as number and percentage. BMI, body mass index. WBC, white blood cell. CRP, c-reactive protein. CKD, chronic kidney disease. COPD, chronic obstructive pulmonary disease.

## Discussion

Prostatic abscess is an uncommon urinary tract infectious disease, making up approximately 0.5% of all urological diseases [16]. If not properly treated, prostatic abscess may lead to severe urosepsis and death. In our study, 12.4% of patients who were hospitalized and diagnosed with prostatitis had prostatic abscess. Based on previous publications, Ha (2008) and Lee (2016) reported the incidence of prostatic abscess among acute prostatitis being 6% and 21.8% separately [2,3]. In a recent population based trial in the U.S., the incidence rate of abscess among hospitalized patients was 5.4% [18]. Prior urinary tract manipulation was a risk factor for development of prostatic abscess [3]. However, in our study, the percentage of patients receiving previous urological procedures was low (lithotripsy 1.0%, TURP 2.0%), and it also showed no correlation with prostatic abscess-related death.

Age was a prominent risk related to death in our study (OR 1.18, p = 0.002). The mean age of patients with prostatic abscess was 68.8 years. Patients in the mortality group were much older (85.1 years) than the survivor group (67.2 years). There were few studies regarding the impact of age on prostatic abscess related mortality. One study comparing treatment methods briefly mentioned 2 deaths in the trial might be related to relatively old-age (71 and 76 years old) [11]. Also, Reddivari AKR (2023) classified age above 65 years old as one poor prognostic factor [16].

Fever, defined as a body temperature higher than 38°C, is also considered a sign of poor prognosis [16]. Considering that hypothermia isa marker of severe infection and has been linked to increased infection-related mortality,.we included both fever and hypothermia in our analysis [19–21]. Consistently, though not reaching statistical significance, more patients in the mortality group had fever or hypothermia at the time of admission (88.9% vs 55.9%, p = 0.074).

Older age was generally thought as a risk factor for benign prostate hyperplasia onset and progression [22]. With the older population in the mortality group, it was reasonable to observe more percentage of alpha blocker use (66.7% vs 21.5%, p = 0.008) in this population. Though initially appearing significant in relation to prostatic abscess related death in univariate analysis, alpha blocker use had shown no increased risk in multivariate study. Instead of a factor directly affecting mortality in prostatic abscess, it was more likely to be a marker of an older and comorbid population. The use of 5 alpha reductase inhibitors was relatively low in our study, reflecting different clinical practice in our region [23].

The most common site of abscess formation in addition to prostatic abscess were liver (50%), followed by kidney (28.5%) in our study. The result was similar to the previous study with 32 prostatic abscess, as 2 liver abscess, 1 renal abscess and 1 buttock abscess were identified among 4 patients with distant spread of abscess [2]. We had found no relation of additional abscess and mortality risk in our study. However, we identified that patients with multi-organ abscess demonstrated a significantly higher probability of undergoing prostatic abscess drainage (OR=3.89, p = 0.035). This finding suggested that multi-organ involvement may reflect a greater disease burden or more extensive infection spread, prompting clinicians to adopt a more aggressive interventional approach for source control. A review of intraabdominal abscess also supported that multiple abscesses being one factor associated with fatal outcome, and ineffective drainage raised the risk of mortality [24].

Conservative treatments can be adopted for smaller abscesses, while drainage for prostatic abscess more than 2 cm was suggested [7]. Surgical intervention had shown to shorten antibiotics duration and decreased hospital stay length [2,16], and the intervention rate of prostatic abscess in previous studies ranged between 56.8% and 60.9% [7,10,14]. Frequent sepsis development (73%) and higher mortality rate were observed in patients with abscess larger than 2 cm, and transurethral abscess unroofing was found to reduce mortality and chronic complications [25]. Regarding the decision to perform drainage for prostatic abscess, one retrospective study attempted to develop a complexity score; however, evidence supporting the benefits of drainage is largely derived from case reports with limited sample sizes [26–28]. In our study, 12 patients had received TURP, 3 transperineal drainage, and one suprapubic prostatectomy. No significant association was observed between drainage procedures and prostatic abscess related mortality or length of hospital stay. Likewise, when TURP was analyzed separately as a specific drainage modality, it showed no significant correlation with clinical outcome either. The lack of association between drainage and survival in our study may be attributed to confounding by indication and the small sample size.

The decision of drainage in our study was based on clinical judgment, considering patients’ general conditions, presence of urinary tract symptoms, experience of radiologist and available manpower.In addition to abscess size, patient performance status, severity of infection, and the need to obtain microbiological cultures are also important considerations in decision-making. In our analysis comparing drainage or TURP with conservative treatment, we identified that prostate volume and cystostomy use were associated with a higher likelihood of undergoing drainage or TURP. These factors may reflect more severe lower urinary tract symptoms, thereby potentially affecting clinicians’ decisions regarding drainage. Interestingly, we found that liver cirrhosis was associated with a higher likelihood of undergoing TURP. Patients with liver cirrhosis are known to have relative immune dysfunction, which predisposes them to severe infections and sepsis [29]. In this context, clinicians may favor more aggressive source control, such as TURP, to achieve better infection control despite the recognition that cirrhosis itself is associated with increased perioperative risk and higher mortality [30].

Propensity score matching was conducted using abscess size, age and body mass index to reduce confounding between drainage or not. Importantly, markers of infection severity, including WBC, CRP and presence of fever were comparable between the drainage and non-drainage groups after matching, probably suggesting similar baseline inflammatory status. In the matched cohort, length of hospital stay did not differ between groups, and the number of mortality events was too small to allow meaningful assessment of the effect of drainage on survival. These findings indicated that, in patients with comparable age, abscess burden, and infection severity, no clear difference in short-term outcomes between drainage strategies could be demonstrated. Further studies with a larger sample size and more descriptive measures of infection severity such as sequential organ failure assessment (SOFA) score are warranted to better delineate which subgroup of patients derive benefits from drainage.

There were several limitations in our study. First, the retrospective, single-center design introduced selection bias and constrained data availability, which limited the generalizability of the findings. Important factors potentially associated with mortality, such as antibiotic regimens and detailed measures of infection severity, could not be fully assessed. Furthermore, although most variables had a high data completeness rate, there was a higher percentage (24.5%) of missing BMI data. Second, the small number of mortality events limited statistical power and restricted the reliability of logistic regression analysis, increasing the risk of model overfitting and unstable estimates. Third, the decision to perform drainage was influenced by multiple clinical factors that were also associated with mortality, resulting in confounding by indication, which complicated interpretation of the association between drainage and survival despite attempts at statistical adjustment. Studies with larger sample sizes and prospective design may be needed to further investigate factors that affect mortality in prostatic abscess, and patients who may benefit from interventions. Despite its limitations, our study of 102 confirmed cases represents one of the larger cohorts and still provides valuable clinical insights into the characteristics and outcomes of patients with prostatic abscess.

## Conclusion

In this retrospective study, older age was the strongest predictor of mortality in patients with prostatic abscess. While larger abscess size and the use of cystostomy were related to a higher likelihood of intervention, they were not associated with survival in our cohort. Drainage also showed no significant association with mortality, possibly due to the limited number of events and potential confounding factors. The benefits of drainage, particularly in elderly patients with prostatic abscess, warrant further investigation.

## Supporting information

S1 Table Characteristics of patient receiving drainage of abscess. This table presents the differences between patients who underwent drainage for prostatic abscess and those who did not.
(DOCX)

S2 Table Characteristics of patients receiving TURP. This table presents the differences between patients who underwent TURP for prostatic abscess and those who did not.
(DOCX)
