# Supplementary material for: Risk factors for mortality in prostatic abscess: Insights into patient characteristics and drainage practices
Source: PLoS One. 2026 Jun 1;21(6):e0349673. doi: 10.1371/journal.pone.0349673 (PMC13225434; doi:10.1371/journal.pone.0349673)
Supplement: S1 Table — This table presents the differences between patients who underwent drainage for prostatic abscess and those who did not. (DOCX) [file pone.0349673.s001.docx]

**S1 Table. Patient characteristics between drainage and non-drainage group**

|  | Drainage  (n=16) | Non-Drainage  (n=86) | p value |
| --- | --- | --- | --- |
| Age, years  Mean (SD) | 64.2 (8.6) | 69.7 (14.8) | 0.060 |
| BMI, kg/m^2^  Mean (SD) | 25.4 (3.7) | 23.9 (4.0) | 0.084 |
| Fever or hypothermia (%) | 8 (50.0) | 49 (57.0) | 0.606 |
| WBC, /µL  Mean (SD) | 15041.9  (8937.4) | 15915.8  (7943.6) | 0.765 |
| CRP, mg/dL  Mean (SD) | 15.4 (9.9) | 15.4 (10.2) | 0.969 |
| Abscess size, cm  Mean (SD) | 4.1 (1.9) | 2.9 (1.2) | 0.014* |
| Abscess at another organ (%) | 5 (31.3) | 9 (10.5) | 0.042* |
| Prostate volume, cm^3^  Mean (SD) | 72.5 (49.7) | 51.2 (30.7) | 0.013* |
| Alpha blocker (%) | 4 (25.0) | 22 (25.6) | 1.000 |
| Cystostomy (%) | 6 (37.5) | 10 (11.6) | 0.018* |
| Length of stay, days  Mean (SD) | 20.4 (9.8) | 22.4 (19.6) | 0.410 |
| Death (%) | 0 (0) | 9 (10.5) | 0.480 |
| Comorbidities | | | |
| Chronic kidney disease (%) | 5 (31.3) | 25 (29.1) | 1.000 |
| COPD (%) | 1 (6.3) | 22 (25.6) | 0.112 |
| Cerebrovascular disease (%) | 5 (31.3) | 25 (29.1) | 1.000 |
| Diabetes mellitus (%) | 10 (62.5) | 39 (45.3) | 0.207 |
| Hyperlipidemia (%) | 5 (31.3) | 20 (23.3) | 0.532 |
| Hypertension (%) | 7 (43.8) | 50 (58.1) | 0.287 |
| Ischemic heart disease (%) | 4 (25.0) | 20 (23.3) | 1.000 |
| Liver cirrhosis (%) | 3 (18.8) | 5 (5.8) | 0.109 |

This table demonstrates different characteristics between drainage and non-drainage group.

Mann-Whitney U-test. Chi-Square test. Fisher’s exact test. *p<0.05, **p<0.01. Continuous data are expressed as mean (standard deviation). Categorical data are expressed as number and percentage. BMI, body mass index; WBC, white blood cell count; CRP, c-reactive protein; COPD, chronic obstructive pulmonary disease.
