# Supplementary material for: Risk factors for mortality in prostatic abscess: Insights into patient characteristics and drainage practices
Source: PLoS One. 2026 Jun 1;21(6):e0349673. doi: 10.1371/journal.pone.0349673 (PMC13225434; doi:10.1371/journal.pone.0349673)
Supplement: S2 Table — This table presents the differences between patients who underwent TURP for prostatic abscess and those who did not. (DOCX) [file pone.0349673.s002.docx]

**S2 Table. Patient characteristics between TURP and non-TURP group**

|  | TURP  (n=12) | Non-TURP  (n=90) | p value |
| --- | --- | --- | --- |
| Age, years  Mean (SD) | 65.1 (8.1) | 69.3 (14.7) | 0.178 |
| BMI, kg/m^2^  Mean (SD) | 25.91(4.3) | 24.0 (4.0) | 0.284 |
| Fever or hypothermia (%) | 7 (58.3) | 50 (55.6) | 0.856 |
| WBC, /µL  Mean (SD) | 13754.2(8061.2) | 16048.7(8073.7) | 0.496 |
| CRP, mg/dL  Mean (SD) | 15.6 (10.8) | 15.3 (10.1) | 0.975 |
| Abscess size, cm  Mean (SD) | 4.0 (1.9) | 2.9 (1.3) | 0.039* |
| Prostate volume, cm^3^  Mean (SD) | 72.4 (56.6) | 52.2 (30.6) | 0.081 |
| Alpha blocker (%) | 2 (16.7) | 24 (26.7) | 0.726 |
| Cystostomy (%) | 5 (41.7) | 11 (12.2) | 0.020* |
| Length of stay, days  Mean (SD) | 19.5 (10.0) | 22.4 (19.2) | 0.713 |
| Death (%) | 0 (0) | 9 (10.0) | 0.594 |
| Comorbidities | | | |
| Chronic kidney disease (%) | 4 (33.3) | 26 (28.9) | 0.744 |
| COPD (%) | 1 (8.3) | 22 (24.4) | 0.289 |
| Cerebrovascular disease (%) | 5 (41.7) | 25 (27.8) | 0.329 |
| Diabetes mellitus (%) | 8 (66.7) | 41 (45.6) | 0.169 |
| Hyperlipidemia (%) | 5 (41.7) | 20 (22.2) | 0.161 |
| Hypertension (%) | 6 (50.0) | 51 (56.7) | 0.662 |
| Ischemic heart disease (%) | 4 (33.3) | 20 (22.2) | 0.470 |
| Liver cirrhosis (%) | 3 (25.0) | 5 (5.6) | 0.050 |

This table demonstrates different characteristics between TURP and non-TURP group.

Mann-Whitney U-test. Chi-Square test. Fisher’s exact test. *p<0.05, **p<0.01. Continuous data are expressed as mean (standard deviation). Categorical data are expressed as number and percentage. TURP, transurethral resection of prostate; BMI, body mass index; WBC, white blood cell count; CRP, c-reactive protein; COPD, chronic obstructive pulmonary disease.
